# When care situations evoke difficult emotions in nursing staff members: an ethnographic study in two Norwegian nursing homes

**DOI:** 10.1186/s12912-015-0093-7

**Published:** 2015-07-30

**Authors:** Anne Marie Sandvoll, Ellen Karine Grov, Kjell Kristoffersen, Solveig Hauge

**Affiliations:** Faculty of Health Studies, Sogn og Fjordane University College, Førde, Norway; Faculty of Health and Sport Sciences, University of Agder, Kristiansand, Norway; Faculty of Health and Social Studies, Telemark University College, Porsgrunn, Norway

**Keywords:** Aversion, Emotions, Ethnography, Nursing homes, Practice

## Abstract

**Background:**

Caring practice in nursing homes is a complex topic, especially the challenges of meeting the basic needs of residents when their behaviour evokes difficult emotions. Cognitive and physical changes related to aging and disability can contribute to behaviours considered to be unacceptable. For example, resident behaviours such as spitting, making a mess with food or grinding teeth are behaviours that most people do not want to see, hear or experience. The aim of this study was to gain a deeper understanding of how nursing home staff members deal with such behaviours in care situations.

**Methods:**

This article draws on ethnographic data to describe how nursing home staff members manage unpleasant resident behaviours. The study was based on two long-term units in two Norwegian public nursing homes. The Region’s Medical Ethics Committee and the Norwegian Social Science Data Services granted approval. In total, 45 participants (37 nursing aides and eight nurses) agreed to participate in this study. Ten of the participants were interviewed at the end of the field study.

**Results:**

This study indicates that nursing home staff members experience difficult emotions related to some residents’ behaviours. However, they found these feelings difficult to express and rarely verbalized them openly. In addition, they were characterized by a strong obligation to help all residents, despite their own feelings. Therefore, it appears that an inner struggle occurs as a part of everyday practice.

**Conclusions:**

Despite these difficult emotions, nursing staff members believed that they needed to manage their responses and continued to offer good care to all residents. These findings extend our understanding of this unarticulated part of nursing home practice.

## Background

Here we report findings from an ethnographic study of nursing home practice, which is often described as complex, [1, 2] even though it might be regarded as consisting of common everyday activities [3]. However, the complexity of nursing home practice is closely related to the challenge of helping frail and sick older people to meet their basic physical, psychosocial and spiritual needs. While nursing staff members consider assisting nursing home residents to be difficult and demanding work [4, 5, 3, 6], the practice and skills needed to accomplish this work are taken for granted [7, 8].

Most residents in nursing homes suffer from cognitive and physical impairments associated with ageing and disability. This might contribute to behaviours considered to be socially unacceptable, such as incontinence and assaulting staff members. Furthermore, the loss of control over bodily functions renders routine social interactions difficult and can be potentially embarrassing and unpleasant for others. We focus on one particularly demanding part of nursing home practice; namely, the issue that the daily work involves ‘annoyances’ from residents who are agitated and those who continually repeat specific words or phrases, actively resist, yell or complain constantly [4, 3]. Soiling can also be a problem when a resident spits or makes a mess with food, and residents might display annoying behaviours, such as grinding their teeth. These are unpleasant behaviours that people in nearly all cultures do not want to see, hear or experience [9, 10]. Nursing tasks dealing with others’ bodies and bodily excretions have been categorized as ‘dirty work’ [11–14].

Emotions such as disgust, contempt and aversion to the physical work involved in nursing homes often remain concealed. However, different theorists have shaped our understanding of disgust or aversion accompanying the physical care of ageing and seriously ill populations [15, 14]. The private activities of the body are normally undertaken by the individual and concealed in the home [14]. The loss of autonomy in these matters constitutes a major social loss and can lead to estrangement or rejection [15]. Van Dongen [16] questions whether it is always correct to conceal one’s emotions from residents and suggests that this should be discussed. However, there is a lack of suitable language for articulating how clinicians perform these tasks [14, 17]. Nursing home staff members deal with difficult behaviours and emotions daily, probably involving both personal morality and the understanding that all humans are of equal value, as well as understanding the importance of treating all residents equally [16, 18]. However, how staff members experience behaviours that might evoke aversion is a part of nursing home practice that is described only rarely [19, 20]. Nursing staff members are trained to perform hygienic practices to prevent infections and disease [21–23]. When dealing with behaviour that deviates from normal practice, a common solution is to prevent soiling or to isolate those residents who practise such behaviours during meals [24]. These responses indicate that the problem is difficult to talk about because of its association with a frail group of patients. Therefore, studies are needed to explore how nursing home staff members deal with these behaviours in care situations, with the aim of gaining a deeper understanding of everyday nursing practices and how they are managed.

## Methods

An ethnographic design was selected for this study to allow an in-depth understanding of daily nursing practice in nursing homes [25]. Ethnographic approaches can help us to comprehend difficult situations, such as behaviours that make care situations challenging and that are not amenable to evaluation with surveys or interviews [26]. Fieldwork is an appropriate approach to exploring an area of nursing practice that is accepted tacitly and difficult to discuss, because the researcher spends time with the informants [25]. Furthermore, ethnographic studies are well suited for learning and advancing thinking of how unexpected situations in nursing practice are interpreted, experienced or understood [27]. This paper is part of a larger project that aims to describe and explore some characteristics of the implicit aspects of nursing home practice, as already reported [28, 29]. It was important to explore and describe the informants’ views of their everyday practice [25]. We were not focused on difficult emotions such as aversion when entering this field; it became important for us to investigate further when it appeared that such emotions were a challenge for staff members. This is a common experience in ethnographic research; a study might be well advanced before the researcher discovers what the research is ‘really’ about [25].

### Context and ethical considerations

This study was carried out two long-term units in two Norwegian public nursing homes. The first nursing home (NH No. 1) lies in a small town and is a complex building consisting of three units, a cafeteria and a day-care centre. There were 74 residents at the time of the study. Each resident had a private single room with a bathroom, and most of them lived there permanently. The study was conducted in the long-term unit, which had 30 residents and 30 nurses/nursing aides at the time when the data were collected. The second home (NH No. 2) is smaller and located in a rural village in a small municipality. This nursing home has two wards with a total of 46 residents, who also had private single rooms with bathrooms. The ward chosen for the study had 20 residents and 15 nurses/nursing aides.

All the residents were frail, with different physical diseases, and most used wheelchairs for mobility. Because of their poor health, they needed help with their morning hygiene care and during meals and other activities. Approval to conduct observation of the staff members interacting with residents was granted by the Region’s Medical Ethics Committee (REK West, University of Bergen) and The Norwegian Social Science Data Services. Voluntary, informal written consent was obtained from all participants. The research process emphasized the principles of anonymity, protection from harm and proper data storage.

### Participants

Thirty-seven nursing aides and eight nurses agreed to participate in this study, hereafter referred to as the ‘nursing staff members’ or ‘participants’. The participants were all female, 25–60 years of age. Two participants had started work in the nursing home within the previous 2 years; the others had worked there for 10–20 years.

### Data collection

Data were collected by the first author over a six-month period (three months in each nursing home) and comprised observations of the staff members providing care in the two nursing homes, document analysis and audiotaped interviews with ten participants.

Morning care activities and meals that required close contact between residents and nursing staff members were especially important situations for data collection. Moreover, it was important to spend time with the participants to gain their trust and confidence. Therefore, the researcher performed small tasks to enter the group of nursing staff members. Gradually, the researcher became a stricter observer. This process was important to avoid getting too closely involved with the residents and to focus on collecting data. Field notes were taken during observations. Short descriptions were written in the field, and at the end of each day, these were expanded to produce more complete descriptions [25].

At the end of each observation period, five of the participants who served as key informants were interviewed. This allowed opportunities to discuss the situations shared by the participants during the observations. For example, these interviews were used to explore further situations in which behaviours evoked aversion or irritation, which particularly contributed to the collection of the valuable data discussed in this paper. The first author transcribed the interviews verbatim immediately after each interview. Document analyses were also performed during the fieldwork. Care policies and procedures were explored and included in the field notes. The material for analysis covered 111 pages of (single-spaced) field notes and 107 pages of transcribed interviews.

### Analysis

In ethnographic studies, the analysis forms a continuous process rather than separate steps [25, 30]. The first step was to read the data carefully to gain a thorough familiarity with them. Detailed and repeated reading was necessary to look for interesting analytical concepts [25]. The concepts were developed spontaneously, either based on statements from the participants or developed by the researcher based on observations. Interesting concepts were identified, some of which had been noted previously in the field notes; for example, ‘aversion’ and ‘commitment’. In the second step, a detailed examination of the identified concepts was undertaken to clarify and deepen our understanding of each concept [25]. The third step involved an interpretation of the relationships between the concepts. For example, how could we understand the fact that the staff members experienced consciousness of their obligations and felt a strong commitment despite the fact that they experienced aversion towards the residents? In this analytical step, different theoretical perspectives, common-sense expectations and stereotypes played an important role [25]. Gradually, we identified associations between the concepts of ‘aversion’, ‘consciousness’ and ‘commitment’. These were used to develop the main category ‘difficult emotions’, which represented the reactions of the nursing staff members to the different emotions that they experienced in their everyday practice. Researchers always enter the field of research with certain opinions about what it entails. As mentioned earlier, we did not have difficult emotions such as aversion in our focus when we entered the field. Furthermore, our long experience from visiting and working in nursing homes made it challenging to question the obvious findings. Throughout the whole analytical process, we circulated and discussed the concepts and aimed to maintain a reflective research position as a team. By striving to be reflexive along with reading different theories, we finally managed to ‘make sense’ of the data and gained new understandings [25, 31].

## Results

The results of our analysis revealed the main category ‘difficult emotions’, which included the three concepts of aversion, consciousness and commitment.

### Aversion

During fieldwork, the researcher observed several care situations that might evoke aversion or irritation. For example, after several weeks of observing the morning care for residents who repeatedly ground their teeth, the researcher wondered how this behaviour affected the staff members. One of them explained as follows.

*I think that all people do things that irritate us, different things irritate different people, but some kinds of behaviour irritate everyone in a way. For example, one resident and the way he grinds his teeth is very intense … he does it a lot (interview, participant 10).*

Clearly, habits such as grinding teeth might become mentally demanding for nursing staff members, who have to listen to it daily. In close-care situations, this intense habit obviously irritates and fatigues them over time.

*Because sometimes it’s just not your day or you just don’t have the energy (field note 25, participant 7).*

Another resident behaviour that challenged the nursing staff members was spitting. Even though they deal with several unpleasant behaviours daily, this was reported as the most challenging.

*It’s just one thing, and that’s spitting, it’s the most disgusting thing in this job. Otherwise, everything is OK, but spitting makes me react, I really struggle with it.… Faeces don’t affect me at all. Vomit smells … but, no it’s only those globs of spit (interview, participant 6).*

This statement shows how nursing staff members struggle with their own feelings towards residents’ different behaviours. Furthermore, other residents complain that it is disgusting when residents spit wherever they sit, so the staff members feel that their only option is to isolate these residents with troublesome habits from the others. This was clearly a difficult decision for them.

*But of course it’s disappointing that he doesn’t get any offer (of activities) … so he’s not there (in the activity room) because he spits all the time, and the others started to complain because of his disgusting habit (interview, participant 6).*

### Consciousness

The researcher further realized that it was difficult for staff members to express how they were affected by these behaviours; the fact that they had to isolate these residents weighed on their conscience over time. These feelings were not revealed until the end of the fieldwork, when the researcher had gained trust among the nursing staff members.

*I asked her how she felt while performing this morning care. ‘Nothing’, she said, and explained how the resident has the right to have her morning care done (field note 25, participant 7).*

The statement above shows how the participant first tried to explain how she accepted her responsibility to deliver care to all residents, regardless of their behaviour. However, later the same day, she returned to the researcher to explain how she really felt, although this was obviously hard for her.

*She came back to me the same day and said: ‘About what you asked me, what it is like to be in such situations. Well, after a while, it’s obvious we get tired’ (field note 25, participant 7).*

There were other signs that indicated how the residents’ behaviour affected the nursing staff members. For example, the participants lowered their voices when they explained how these behaviours affected them. Furthermore, they did not share with the researcher how difficult these tasks were until the end of the fieldwork or during the interviews, most likely because it was not until then that trust had been established between the researcher and staff members.

When the staff members needed to isolate certain residents daily, it seemed to prey upon their conscience because they knew the residents and their need for activities and social relationships. This seems to have constituted a considerable burden for them over time.

*Unfortunately, he is one of the residents who often sit alone (interview, participant 10).*

It was obvious that the staff members regretted their lack of opportunity and time to offer the same services to everyone and that some residents spent time sitting alone even though the staff members knew that the resident might have preferred to join in social activities. Altogether, dealing with these issues on a daily basis evoked difficult emotions in staff members. These were emotions that they normally kept to themselves, without showing them to the residents or other staff members. This indicates an undeveloped language for acknowledging the work and the complex intersection of social norms.

### Commitment

As described previously, residents displayed different behaviours that obviously challenged the staff members. However, even though they considered it to be demanding or felt aversion when the residents would spit, grind their teeth or display other such behaviours, the staff members simply dealt with it. One participant explained how she dealt with spitting.

*P: But I have to deal with it, I just bring a big towel and dry it up … I just have to bear it*

*I: You just get on with it?*

*P: Yes, I don’t feel that the others should deal with it. I guess they don’t like it either … I do it myself, I just have to manage it (interview, participant 6).*

This shows how the nursing staff members put the residents’ behaviour and needs before their own reactions and feelings, demonstrating their sense of responsibility. Because of their commitment, they did not ask others to perform a task that they considered to be unpleasant but rather did it themselves.

The staff members’ commitment to setting aside their own feelings appeared to be deeply rooted. Our impression was that they were driven by a strong obligation to help residents, despite struggling with their own feelings, an obligation that led them to feel a strong commitment to care for all residents equally.

## Discussion

This study indicates that the nursing home staff members experienced difficult emotions associated with some residents’ behaviours. However, they found these feelings difficult to express and rarely verbalized them openly. In addition, they were characterized by a strong obligation to help all residents, despite their own feelings. Therefore, it appears that an inner struggle occurs as a part of everyday practice.

We mentioned in the Background section that this combination of aversion and obligation has been described only rarely. It took considerable time and trust before the staff members in our study discussed these issues with the researcher. Their restraint confirmed that it was difficult to talk about this issue, which is consistent with previous research [14, 19]. Van Dongen’s study also revealed that deeper probing regarding the subject of ‘body work’ brought staff members’ emotions to the surface [16]. These difficult emotions also included being concerned about residents sitting alone, as discussed by Kayser-Jones [32]. There might be aspects of nursing staff members experience that - if expressed- could be shared; however, staff members always know more than they can articulate, as discussed by Clinton [33].

Even though they experienced aversion or irritation, the participants in our study tried to deal with this work as best they could, regardless of how they felt. This is in line with Twigg [11], Van Dongen [16] and Lawler [14], who showed how staff members’ feelings about - for example sputum - are determined, and how this is just something that ‘has to be done’. Furthermore, their emotions were concealed from the patients [16, 14]. It seems likely that the nursing staff members in our study felt that they would compromise their professional values if, for example, they asked someone else to deal with a resident’s sputum. Stacey [13] showed how nursing staff members draw meaning from their willingness and ability to perform dirty tasks that others avoid, because they know that it improves the residents’ lives. Twigg [11] showed how care workers accept dealing with dirty work and that they have to suppress any sense of disgust.

Our study revealed that it is important for nursing home staff members to manage their work in ways that maintain complex norms of social comportment. This is in line with Häggström et al. [34], who found that caregivers’ commitment to their relationships with older people is strong. The staff members’ commitment to the residents is also shown in the difficult emotions revealed when, for example, they have to isolate residents because of their unpleasant behaviour. Staff members reported feeling badly about not having the time to offer the same activities to everyone, although they try their best to meet all residents’ needs, as previously discussed [28, 35].

The staff members interviewed in this study appeared to experience a strong commitment to the residents even though residents’ behaviours sometimes evoked difficult emotions. This strong commitment to help the residents can be interpreted as being incorporated into the habitus of staff members over time. According to Bourdieu [36], habitus is the product of history; it establishes individual and collective practices and is often taken for granted within a group [8]. A substantial component of the health revolution was the socio-cultural transformation in personal hygiene and cleanliness [21, 22], which has been incorporated into the habitus of staff as a basic part of nursing [23]. Therefore, when nursing staff members have to deal with residents who deviate from this assumed hygienic practice, dealing with the behaviour itself is difficult, as discussed by Lawler [14]. According to Bourdieu, members of a group act according to practical sense, and when asked to describe the rules with which they comply, they are unable to do so [36]. A larger set of rules and norms are being enacted that may be very difficult for staff members to identify and express fully, even when they are invited to do so.

## Conclusions

Nursing staff participants reported that close contact with some residents’ behaviours could evoke difficult emotions, such as irritation and aversion. However, it was difficult for them to express these feelings, which would have weighed on their conscience when they were constrained to isolate residents with unpleasant behaviour. Despite this, nursing staff members seemed to be committed to managing their responses and to offering good care to all residents.

### Strengths and limitations of the study

One limitation of this study is that it was carried out in a Norwegian context and so might not be generalizable to other countries and cultures. Another limitation is that the data were based on the observations of one researcher. Participant observation is subjective and will always be coloured by the researcher’s expectations. Therefore, during the whole process, including data collection, analysis and interpretation, discussions were carried out with the co-authors. Furthermore, we endeavoured to gain trust and acceptance among the nursing staff members. This involved hard work and a patient process of gaining, building and maintaining mutual trust and acceptance between the researcher and the participants. Thereafter, they opened up and talked about these demanding tasks. The data that we have gathered would have been difficult to obtain with surveys or interviews.

### Implications for practice and research

We have discussed how nursing staff members dealt with aversion to behaviours that most people do not want to see, hear or experience. By focusing on this tacit issue, we made this problem visible, enabling its description and discussion. Staff members found it difficult to express the fact that these behaviours evoke emotions such as irritation and aversion. It is probable that the emotions discussed here have an impact on the relationships between staff and residents and are important for the well-being of everyone, in addition to their impact on the quality of care. Therefore, these matters should be explored further. Moreover, it is likely that our study only revealed a small part of the difficult and tacit tasks undertaken in nursing homes, and this suggests that further research is required to understand the patterns of care that are unspoken and taken for granted.
